# Hepatitis B vaccination coverage among Indian dental students – Systematic review and meta-analysis

**DOI:** 10.1016/j.pmedr.2026.103691

**Published:** 2026-11-01

**Authors:** Deepika Chenna, Abhinav N. Tadikonda, Mithun Pai, Maanissa Phukan, Danica Alicia Menezes, Kalyana C. Pentapati

**Affiliations:** a Department of Immunohematology and Blood Transfusion, Kasturba Medical College, Manipal Academy of Higher Education, Manipal, India; b Department of Public Health Dentistry, Sri Sai College of Dental Surgery, Vikarabad, Telangana, India; c Department of Public Health Dentistry, Manipal College of Dental Sciences Mangalore, Manipal Academy of Higher Education, Manipal, India; d Department of Public Health Dentistry, Manipal College of Dental Sciences, Manipal Academy of Higher Education, Manipal, India

**Keywords:** Compliance, Hepatitis B, Vaccination, Dental, Students, India, Quality education, Good health and well being

## Abstract

**Objective:**

To estimate the pooled estimates of hepatitis B vaccination (HBV) coverage among Indian dental students.

**Methods:**

This systematic review was conducted in accordance with the PRISMA guidelines. The protocol was registered in PROSPERO. A systematic search was conducted in PubMed (1946 onward), CINAHL (1937 onward), Dentistry and Oral Sciences Source (journal-specific coverage), Embase (1947 onward), Web of Science (1900 onward), and Scopus (1970 onward) till July 1st, 2025. Cross-sectional studies conducted among Indian dental students that reported HBV coverage and studies reported in English were included. The meta-analysis was performed via OpenMeta [Analyst] (random effects model with the restricted maximum likelihood method).

**Results:**

A total of 24 studies that reported the HBV were included. (total sample: 8740 (age range: 18–27 years). The pooled estimate of complete HBV coverage was 53.9% (95%CI: 41.84, 65.97), with high heterogeneity (I^2^ = 99.7). Monitoring of the antibody titer was reported in only 5 studies (*n* = 504). Egger's regression revealed no publication bias (Z = 1.73; *P* = 0.08). Meta-regression revealed no significant effect of age or sex distribution on the estimates of HBV coverage. However, studies on clinical dental students reported higher estimates than studies that combined preclinical and clinical students (coefficient: 0.37; *P* < 0.01).

**Conclusion:**

Approximately half of the dental students had not completed the three-dose HBV.

## Highlights

• The complete vaccination for hepatitis B was sub-optimal among dental students.
• There was no association of demographic factors with Hepatitis B vaccination.
• Clinical students had higher estimates than both preclinical and clinical students.

## 1 Introduction

Hepatitis B infection remains a significant global problem. Dental students are at high risk of communicable diseases such as viral hepatitis and HIV due to their close contact with saliva and body fluids and their ability to handle sharp instruments. Both of these infections are serious, highly infective and can cause serious complications (Singh et al., 2011). Coupled with the infectious nature of these diseases, a lack of knowledge regarding transmission routes and universal precautions can be a serious occupational risk for this vulnerable group during their training years (Kumar et al., 2015; Maroof et al., 2012; Sharma et al., 2019; Singh et al., 2011). Although hepatitis B is highly infectious, it can be prevented by prophylactic vaccination. Hepatitis B vaccinations (HBV) are easily available for all age groups with two different regimens, viz., 0, 1 and 6 months or 0, 1 and 2 months. Both of these regimens are effective, and the latter is recommended for rapid protection (Hepatitis B Foundation, 2022).

The risk of hepatitis B infection among health care workers is four times greater than that in the general population (Dannetun et al., 2006). It is estimated that 40% of healthcare workers in developing countries are infected with Hepatitis B (Prüss-Üstün et al., 2003). Nikolopoulou et al. reported global burden of hepatitis B infection among healthcare workers. In Europe and some of the Asian countries it ranged from 0.6 to 1.2%. However, higher prevalence rates of 9.85% were seen in other countries of Asia and Africa (2.3–11.8%). Higher than 8.7% prevalence rates were reported from countries like Cameroon, Mauritania, Sierra Leone, and Tanzania (Nikolopoulou et al., 2023).

Compliance of HBV among dental students was very low (<50%) on the basis of existing studies on HBV coverage among Indian dental students (Benarji et al., 2021; Breena et al., 2019; Khandelwal et al., 2018; Kumar et al., 2015; Mohan et al., 2024; Pratha & Geetha, 2017; Rami et al., 2024; Rathor et al., 2019; Sharma et al., 2019; Shashikala & Chowdhary, 2024). A previous meta-analysis of medical students reported a low HBV rate among South Asian medical students (Lamichhane et al., 2025). Rahmani et al. reported the complete HBV coverage in the range of 40.3 to 100.0% among health care students and residents globally in highly developed countries (Rahmani et al., 2022). Previous studies from developing countries have reported wide variability in HBV coverage. Complete HBV coverage ranged from 1.9%–21.5% (Amirzade-Iranaq et al., 2020; Inthakoun et al., 2024; Nasr et al., 2024; Yildirim et al., 2017).

The Occupational Safety and Health Administration (OSHA, 2026.) and CDC (Centers for Disease Control, 2012) have mandated HBV for all health care professionals who are in close contact with blood or other potential infectious materials. Regulatory bodies in India (Dental Council of India) have not mandated HBV for dental students (Dental Council of India, 2007).

Several systematic reviews and meta-analyses (SRMAs) have been conducted on the prevalence of hepatitis B infection among the general population from various countries (Alali & Abo-Shehada, 2022; Kilonzo et al., 2024; Madihi et al., 2020; Schweitzer et al., 2015; Tadesse et al., 2025). There is a gap with respect to the data on HBV coverage (Lamichhane et al., 2025; Madihi et al., 2025)^,^ and only one SRMA was conducted on HBV among medical students in Southeast Asia (Lamichhane et al., 2025). There was no SRMA on HBV coverage among dental students. Considering the above, epidemiological data on HBV coverage among high-risk health care students are needed. Hence, this SRMA aimed to evaluate pooled estimates of HBV coverage among Indian dental students and explore the potential mediators that may influence HBV coverage.

## 2 Methods

This systematic review was conducted in accordance with the PRISMA guidelines. The protocol was registered in PROSPERO (CRD420251116789) (PROSPERO, 2025). No amendments were made after the preparation of the protocol.

### 2.1 Search

A systematic search was conducted in six electronic databases: PubMed/MEDLINE (1946 onward), Scopus (from inception, with extensive coverage from 1970 onward), Embase (1947 onward, including Embase Classic), CINAHL (1937 onward), Dentistry & Oral Sciences Source (from inception, with journal-specific coverage), and Web of Science Core Collection (1900 onward) until 1st July 2025.

There were no date restrictions. A combination of keywords “dental students”, “Hepatitis B vaccination”, and “India” were used. The search strategy for EMBASE was “(‘dental student’/exp OR ‘dental student*’:ti,ab,kw OR ‘dental undergraduate*’:ti,ab,kw OR ‘dental intern*’:ti,ab,kw OR ‘dentistry student*’:ti,ab,kw OR ‘BDS student*’:ti,ab,kw OR ‘dental college*’:ti,ab,kw) AND (‘hepatitis b'/exp OR ‘hepatitis b vaccine’/exp OR ‘hepatitis B':ti,ab,kw OR ‘HBV’:ti,ab,kw) AND (‘vaccination’/exp OR ‘vaccin*’:ti,ab,kw OR ‘immuniz*’:ti,ab,kw OR ‘immunis*’:ti,ab,kw)”. Backward citation search was performed manually. The reference lists of all the included publications were checked to identify eligible studies.

### 2.2 Selection criteria

Cross-sectional studies that reported the HBV coverage among Indian dental students and those reported in English were included. Non-English studies were out of the scope of this review as we limited the studies that were conducted among Indian dental students. Studies reported in other languages, brief reports, commentaries or conference abstracts or preprints or grey literature were excluded. Studies that did not report separate estimates for dental students were also excluded.

### 2.3 Screening

The search results were added to Rayyan, a web-based interface for screening. Duplicates identified through searches in multiple databases were removed. Titles and abstracts were independently screened by two trained reviewers. The full texts of the included studies were further screened by two trained reviewers independently. Any discrepancies between the reviewers were resolved by the third reviewer. Data extraction included details of the authors, year of publication, sample size, number of students who completed HBV (3 doses), type of student (preclinical, clinical or both), age and sex distribution, and information related to antibody titer screening after vaccination. The primary outcome was complete HBV coverage among dental students who had received all three doses of the hepatitis B vaccine (either 0, 1, and 6 months or 0, 1, and 2 months). HBV coverage was determined based on self-reported information or documented vaccination records, as reported in the included studies. No attempt was made to contact the study authors.

### 2.4 Risk of bias assessment

Risk of bias assessment was performed via the 9-item questionnaire developed for prevalence studies by Hoy et al. (2012). Risk-of-bias findings were summarized descriptively at the domain level.

### 2.5 Statistical analysis

The meta-analysis was performed via the restricted maximum likelihood (REML) method and random effects model because of the high heterogeneity among the included publications. Untransformed prevalence estimates were pooled using REML as it provides a less biased and more efficient estimate than other available methods. Sensitivity analysis was performed via the leave-one-out method. Meta-regression was performed via a mixed methods model. The predictors used were age, sex ratio, and publication year. Subgroup analysis was performed for type of student and geographic zones of India. No distinction was made between self-reported information or documented vaccination records and vaccination schedule in the meta-analysis. Publication bias was estimated via funnel plots and Egger's regression. Heterogeneity was assessed using I^2^ as it is standardized, interpretable, and less sample-size–dependent measure. I^2^ is derived from Cochran's Q statistic adjusted for degrees of freedom. Missing information was not imputed, and all analyses were conducted using available data. The certainty of evidence for the complete HBV coverage was evaluated using a GRADE-adapted approach for prevalence estimates. It included risk of bias, inconsistency, indirectness, imprecision, and publication bias. All the analyses were performed via OpenMeta[Analyst] (for Windows 10) (OpenMeta[Analyst] -- CEBM @ Brown, 1999).

## 3 Results

A total of 442 studies were obtained through a systematic search of six databases, of which 213 were available for title and abstract screening. Full-text screening was performed for 27 studies, and two studies (Karthiga Devi et al., 2020; Madhumitha et al., 2020) were not available. Seven articles were excluded due to missing outcome (*n* = 3), mixed population (*n* = 3) and wrong population (*n* = 1). An additional four eligible studies were identified through backward citation searching. Thus, data extraction was performed for 24 studies [Fig. 1].

**Fig. 1:**
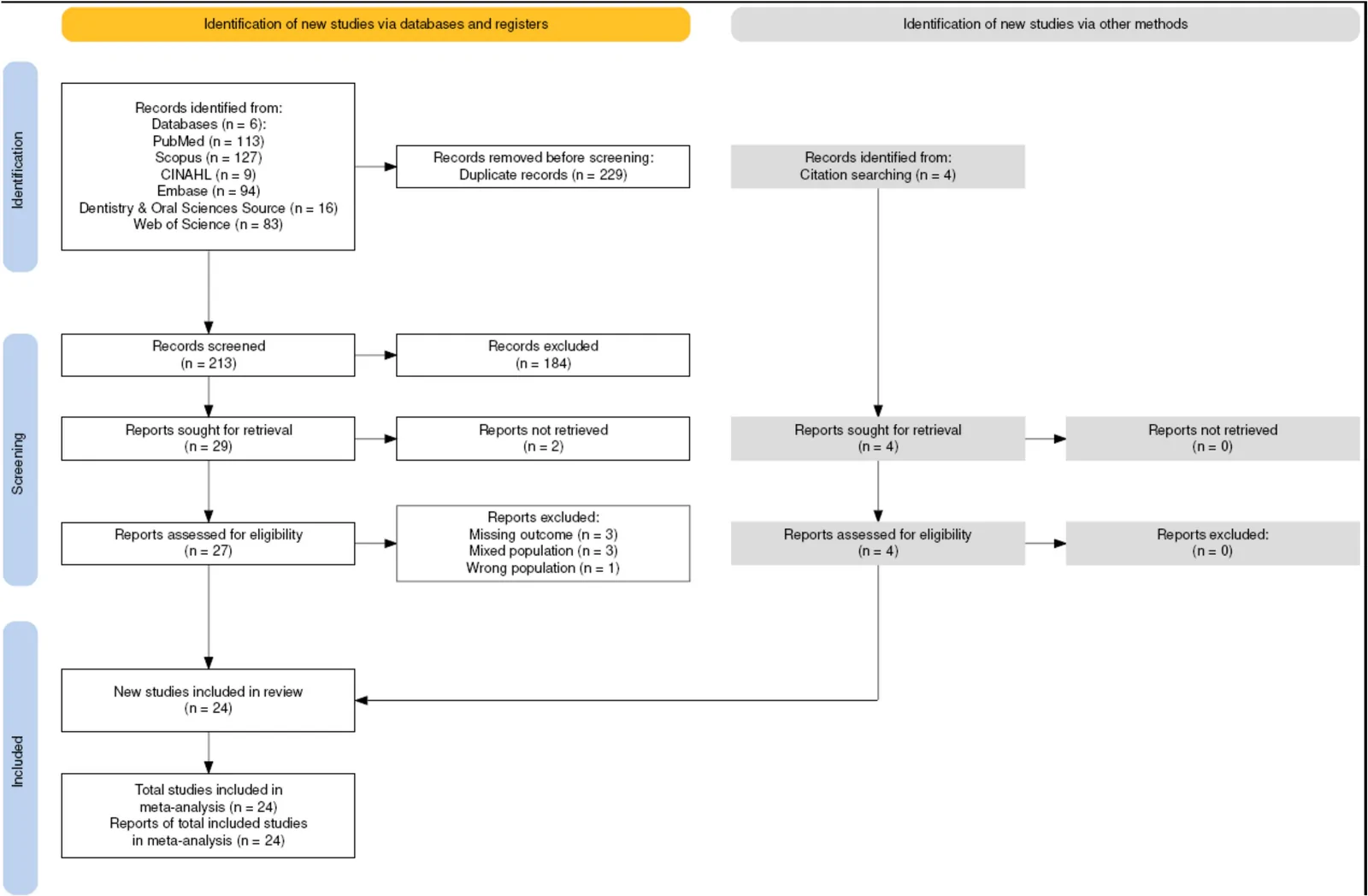
PRISMA 2020 flow diagram illustrating the identification, screening, eligibility assessment, and inclusion of studies in the systematic review and meta-analysis.

### 3.1 Age and sex distribution

Only half of the studies (*n* = 13) reported a mean age of which was 22.1 years. ((Baskaran et al., 2025; Benarji et al., 2021; Breena et al., 2019; Khandelwal et al., 2018; Kumar et al., 2015; Monika Gayathri & Santhosh Kumar, 2016; Nagpal & Hegde, 2016; Rami et al., 2024; Ravoori et al., 2018; Sharma et al., 2019; Shashikala & Chowdhary, 2024; Tirounilacandin et al., 2009; Vasanthakumar & D'Cruz, 2013) A total of 18 studies reported the sex distribution (Baskaran et al., 2025; Benarji et al., 2021; Dubey et al., 2025; Kashyap et al., 2024; Khandelwal et al., 2018; Kumar et al., 2015; Malhotra et al., 2017; Mohan et al., 2024; Rami et al., 2024; Rathinavelu & Sai Pavithra, 2015; Ravoori et al., 2018; Santhosh Kumar, 2016; Sharma et al., 2019; Shashikala & Chowdhary, 2024; Singh et al., 2011; Srinivasan et al., 2022; Sundar Ray et al., 2017; Vasanthakumar & D'Cruz, 2013). A total of 2083 males and 4993 females were included in this review. The average male: female ratio was 0.49 [Table 1].

**Table 1:** Characteristics of the included studies, demographic profiles of participants, and hepatitis B vaccination coverage among Indian dental students.

Author year	Sample size	Mean age	Sex ratio	Complete vaccination coverage (n)	Vaccination Coverage (%)	Student type
Tirounilacandin et al. (2009)	49	24.5		46	93.88	C
Singh et al. (2011)	245		0.35	240	97.96	C
Vasanthakumar and D'Cruz (2013)	179	20	0.46	127	70.95	P
Rathinavelu and Sai Pavithra (2015)	200		0.68	160	80.00	B
Kumar et al. (2015)	216	21.3	0.40	96	44.44	C
Monika Gayathri and Santhosh Kumar (2016)	100	23		62	62.00	C
Santhosh Kumar (2016)	100		1.33	87	87.00	C
Nagpal and Hegde (2016)	486	21.5		311	63.99	B
Pratha and Geetha (2017)	104			39	37.50	C
Khandelwal et al. (2018)	240	23.5	0.82	88	36.67	C
Sundar Ray et al. (2017)	269		0.77	141	52.42	C
Malhotra et al. (2017)	117		0.26	62	52.99	C
Ravoori et al. (2018)	196	22.4	0.56	133	67.86	C
Rathor et al. (2019)	625			274	43.84	C
Sharma et al. (2019)	1200	20	0.16	49	4.08	B
Breena et al. (2019)	300	21		77	25.67	B
Benarji et al. (2021)	2701	21.87	0.50	270	10.00	B
Srinivasan et al. (2022)	84		0.22	70	83.33	C
Shashikala and Chowdhary (2024))	250	23.39	0.35	19	7.60	B
Rami et al. (2024)	229	21.3	0.24	59	25.76	C
Mohan et al. (2024)	182		0.26	7	3.85	B
Kashyap et al. (2024)	243		0.65	218	89.71	C
Baskaran et al. (2025)	200	23.49	0.42	172	86.00	C
Dubey et al. (2025)	225		0.38	152	67.56	C

C: Clinical; P: Preclinical; B: Both preclinical and clinical.

### 3.2 Quality assessment

All studies (100%) were classified as having a high risk of bias concerning national representativeness, while 22 studies (91.7%) were rated as high risk regarding the adequacy of the sampling frame. For sampling methods, 11 studies (45.8%) were classified as high risk, two (8.3%) as low risk, and 11 (45.8%) as unclear risk. Regarding internal validity, nonresponse bias, only eight studies (33.3%) were classified as having a low risk. All studies were rated as low risk for direct data collection. Nine studies (37.5%) had a high risk of bias regarding the definition of HBV, while 12 (50.0%) were classified as low risk and three (12.5%) as unclear risk. The reliability and validity of vaccination-status ascertainment could not be adequately established in any of the included studies. Consistency in data collection was rated as low risk in all the studies. All studies were rated as low risk regarding the appropriateness of the numerator and denominator.

Overall, the findings indicate substantial concerns regarding the representativeness of the study populations, incomplete reporting of nonresponse, and uncertainty regarding the validity of vaccination-status measurements. These limitations should be considered when interpreting the pooled HBV coverage.

### 3.3 Type of student

The majority of the studies (*n* = 16) were conducted among clinical dental students (Baskaran et al., 2025; Dubey et al., 2025; Kashyap et al., 2024; Khandelwal et al., 2018; Kumar et al., 2015; Malhotra et al., 2017; Monika Gayathri & Santhosh Kumar, 2016; Pratha & Geetha, 2017; Rami et al., 2024; Rathor et al., 2019; Ravoori et al., 2018; Santhosh Kumar, 2016; Singh et al., 2011; Srinivasan et al., 2022; Sundar Ray et al., 2017; Tirounilacandin et al., 2009) [Table 1]. Only one study was reported exclusively among preclinical students (Vasanthakumar & D'Cruz, 2013) and was not included in the subgroup analysis. Subgroup analysis revealed that estimates of HBV coverage were greater among clinical dental students (64.4%; 95% CI: 53,76) than estimates from studies conducted among both preclinical and clinical dental students (27.8%; 95% CI: 4.6,51) [Fig. 2].

**Fig. 2:**
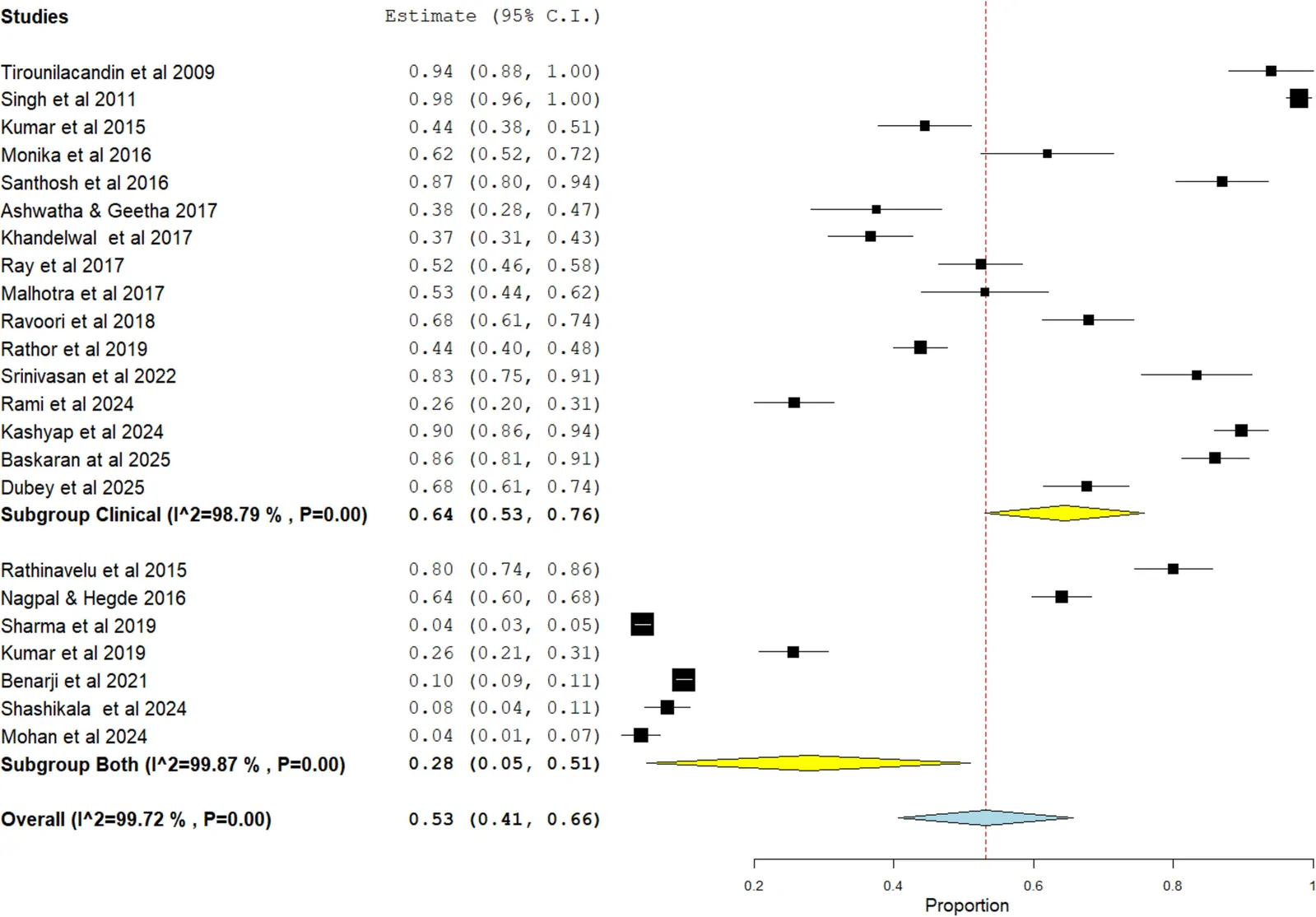
Subgroup analysis of hepatitis B vaccination coverage according to dental student category (exclusively clinical versus combined preclinical and clinical students).

### 3.4 Geographic distribution

Majority of the studies (*n* = 10) were reported from south India with an overall estimate of 65.9%(95% CI: 50,81.8) (Baskaran et al., 2025; Benarji et al., 2021; Monika Gayathri & Santhosh Kumar, 2016; Nagpal & Hegde, 2016; Pratha & Geetha, 2017; Rathinavelu & Sai Pavithra, 2015; Ravoori et al., 2018; Santhosh Kumar, 2016; Tirounilacandin et al., 2009; Vasanthakumar & D'Cruz, 2013). This was followed by west zone with 5 studies (29.1%) (Breena et al., 2019; Rami et al., 2024; Sharma et al., 2019; Shashikala & Chowdhary, 2024; Srinivasan et al., 2022), east zone with 4 studies (47.4%) (Kashyap et al., 2024; Mohan et al., 2024; Rathor et al., 2019; Sundar Ray et al., 2017), central zone with 3 studies (59.8%) (Khandelwal et al., 2018; Kumar et al., 2015; Singh et al., 2011), with only 2 studies from north zone (60.7%) (Dubey et al., 2025; Malhotra et al., 2017) [Fig. 3].

**Fig. 3:**
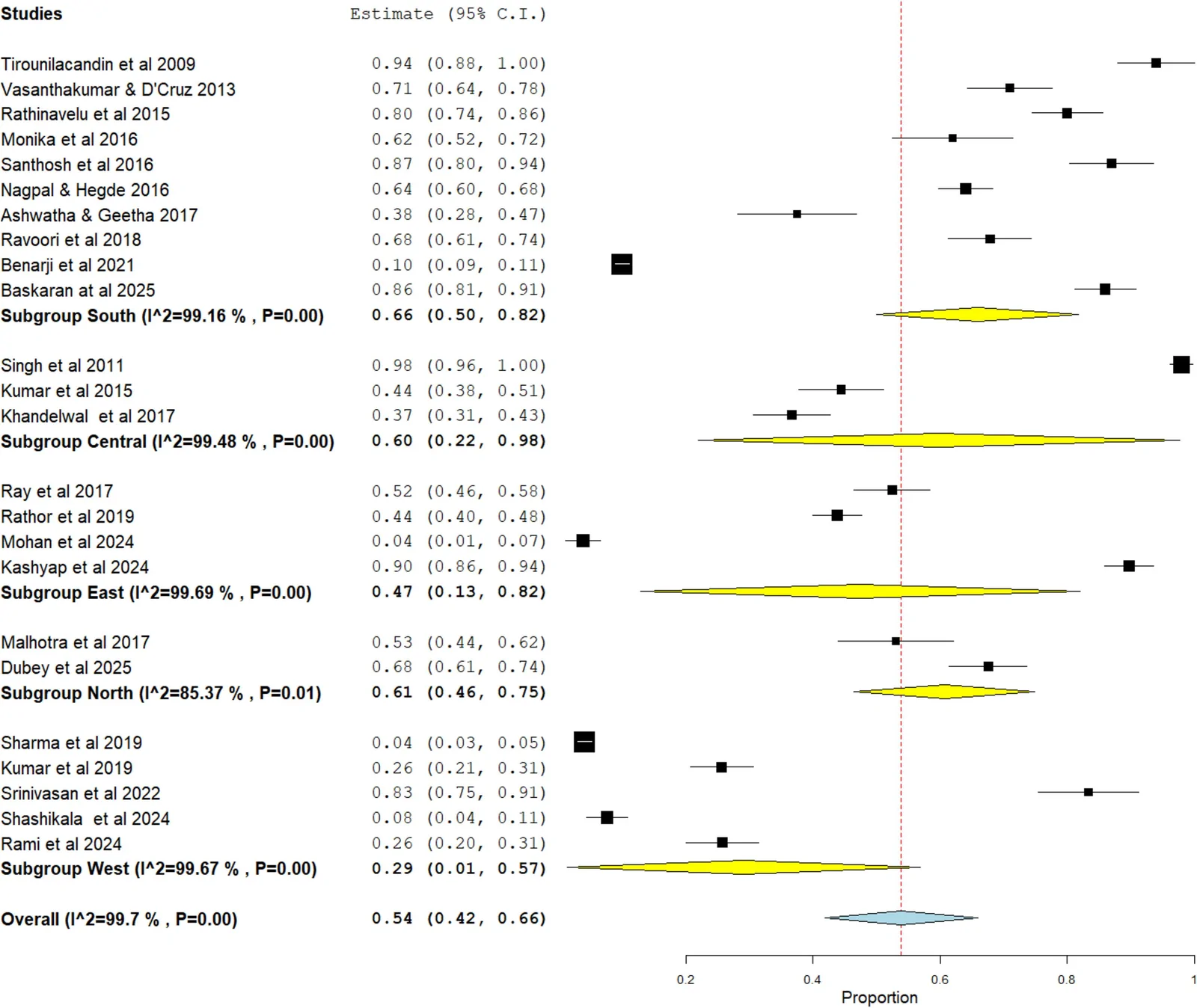
Subgroup analysis of hepatitis B vaccination coverage among dental students according to geographical region in India.

### 3.5 Meta-analysis, meta-regression and subgroup analysis

The sample size ranged from 49 to 2701, with a total sample of 8740. The age ranged from 18 to 27 years. Among the total 8740 subjects, complete HBV coverage was reported by 2959. The pooled prevalence of complete HBV coverage was 53.9% (95% CI: 41.84,65.97), with high heterogeneity among the included studies (I^2^ = 99.7) [Fig. 4]. Monitoring of the antibody titer was reported in only 5 studies (*n* = 504). Meta-regression revealed that age (coefficient: 0.08; 95% CI: −0.03,0.2) and the male:female ratio (coefficient: 0.42; 95% CI: −0.1,0.94) were not associated with overall estimates of HBV coverage. The publication year had a nontrivial negative influence on the overall estimates of HBV coverage (coefficient: -0.03; 95% CI: −0.05,0). Clinical dental students reported higher estimates of HBV coverage than did combined preclinical and clinical dental students (coefficient: 0.37; 95% CI: 0.14,0.6). Sensitivity analysis via the leave-one-out method revealed no major deviations in the overall HBV coverage estimates.

**Fig. 4:**
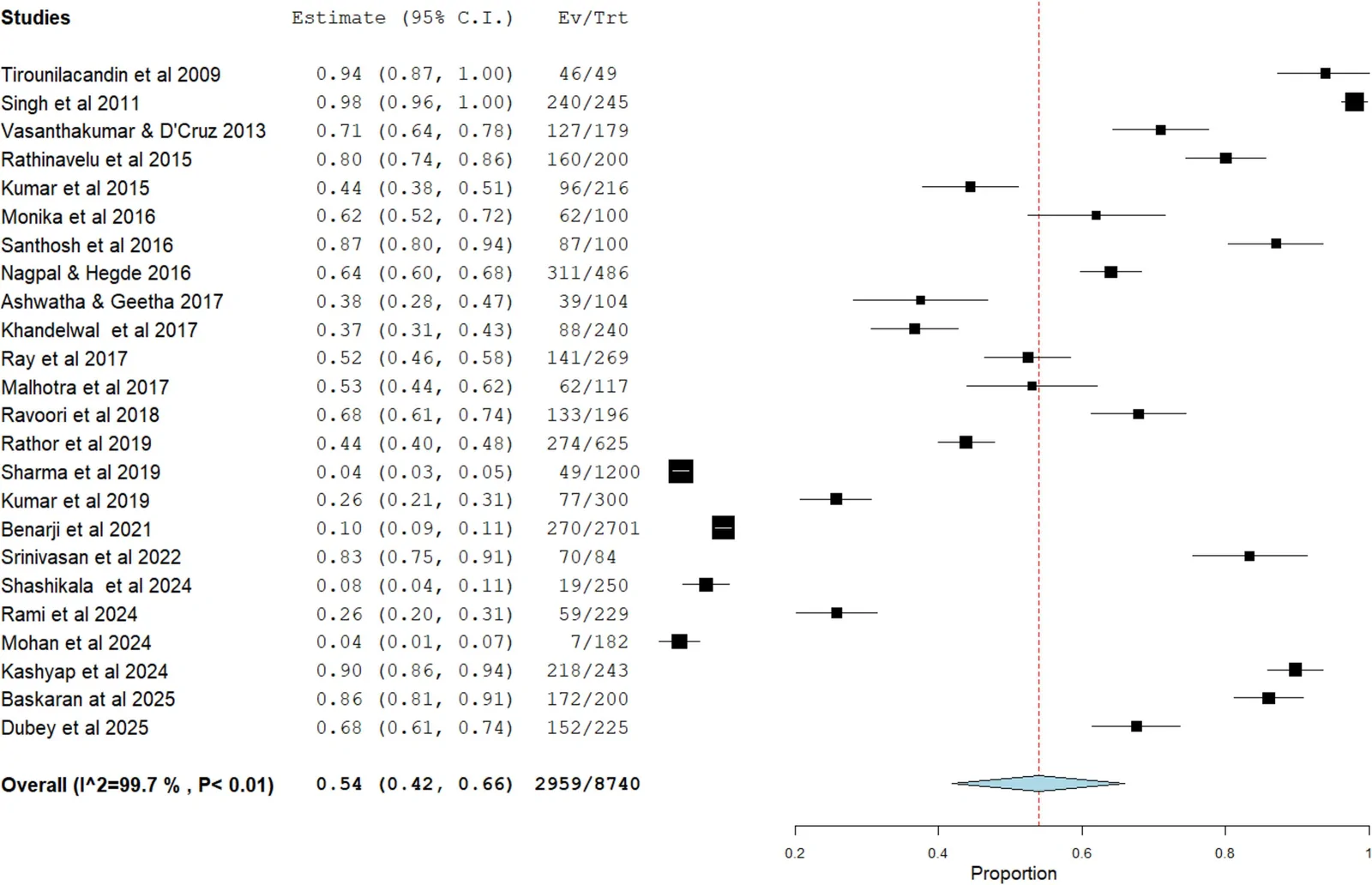
Forest plot showing the pooled prevalence of complete hepatitis B vaccination coverage among Indian dental students.

### 3.6 Publication Bias and certainty of evidence

The funnel plot revealed no asymmetry, and Egger's regression revealed no publication bias (Z = 1.73; *P* value = 0.08) [Fig. 5]. The certainty of evidence for the pooled estimate of complete HBV coverage was very low. The main concerns were limitations in representative sample and sampling methods, substantial heterogeneity, and the relatively wide confidence interval of the pooled estimate. There was no statistically significant evidence of funnel plot asymmetry.

**Fig. 5:**
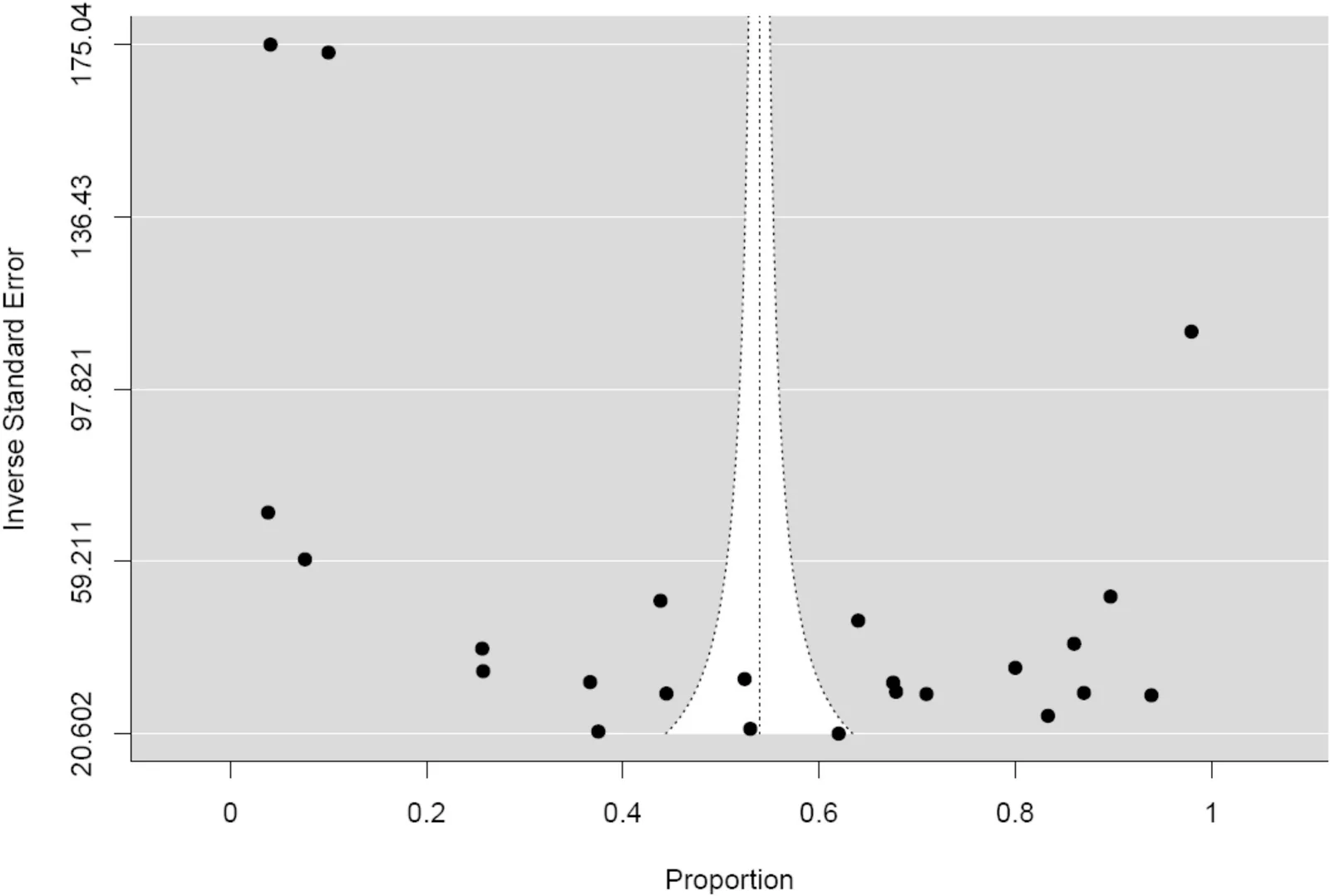
Funnel plot assessing potential publication bias in studies reporting hepatitis B vaccination coverage among Indian dental students.

## 4 Discussion

Future dental professionals are highly vulnerable to the risk of hepatitis B virus infection, and the only effective solution to mitigate this risk is to ensure HBV. HBV is of utmost importance for these young professionals because of their commitment to invasive dental procedures and high risk of needle stick injuries during training years. This would ensure maintenance of good health and wellbeing and quality education aligning with sustainable developmental goals. Understanding the acceptance and compliance of HBV coverage can help us understand and implement effective strategies. Owing to the variations in prevailing policies, guidelines in various countries and vaccine acceptance, we restricted our review to Indian dental students.

We conducted a systematic search of the six most popular databases combined with a search of references included in publications conducted among Indian dental students. A total of 24 studies with a population of 8740 were included. Meta-analysis revealed low HBV (54%) coverage among Indian dental students. The included studies showed high heterogeneity (I^2^ = 99.7), and the results must be interpreted carefully. Similar results were reported in a meta-analysis among medical students from Southeast Asia (Lamichhane et al., 2025). Previous studies have reported wide variability in HBV coverage. An Iranian study reported that only 1.9% of dental students were fully vaccinated (Amirzade-Iranaq et al., 2020). Similarly, a study from Laos found that just 6.3% of dental students had completed the vaccination schedule (Inthakoun et al., 2024). In Pakistan, only 17.2% of medical and dental students reported complete HBV coverage (Nasr et al., 2024). In contrast, a higher coverage of 54.8% was observed among dental healthcare workers in Pakistan, suggesting improved uptake in clinical groups (Kiyani et al., 2020). Likewise, a study from Turkey among dental students reported that only 21.5% were fully vaccinated (Yildirim et al., 2017).

Owing to the high heterogeneity, meta-regression was performed to evaluate the potential predictors that may have influenced the overall estimates. Age and the sex ratio had no significant influence on the overall estimates. The publication year had a small negative influence, whereas the type of student had a significant influence on the overall estimates. This may be due to the possibility of mandatory vaccination implemented in most dental schools during the transition from preclinical to clinical years. Also, the possibility of higher deferral may exist in preclinical dental students due to the low occupational risk perception. Several factors may contribute to this heterogeneity. India has regional diversity in the available healthcare infrastructure, implementation policies, and institutional governance. These factors may also have influence on the vaccination coverage among dental students. Also, the cost, ease of vaccine accessibility, awareness, and availability, may also have role on the vaccination coverage. Variations in public health initiatives and campaigns and accreditation requirements might have amplified the variability in the vaccine acceptance.

Complete HBV does not invariably ensure adequate immunological response among all the individuals. A small proportion of individuals become non-responders or develop suboptimal antibody titers. Due to the high occupational risk in dentistry, reliance on self-reported vaccination coverage without serosurveillance is inadequate. The lack of documented antibody titers can complicate post-exposure management, infection-control protocols and the need for booster doses after occupational hazards.

Our review has several limitations. Studies that were published in the English language were included. Additionally, all the studies were cross-sectional in design, with a lack of appropriate sampling methods, sample size estimation and self-reported vaccination coverage, high heterogeneity, limited reporting of antibody titer data could have impacted the generalizability of the estimates. To address these limitations, sensitivity analysis revealed no potential deviation in the results, and there was no publication bias, as evidenced by Egger's regression test. Due to the primary methodological limitations related to the representativeness and selection of study participants among the included publications, the generalizability of the pooled estimates of HBV coverage is limited.

The potential impact of recall bias and social desirability bias cannot be adequately examined. Individuals may inaccurately recall or misreport their vaccination coverage when not verified using medical records. Given that HBV is perceived as a professional obligation, there is a possibility of overreporting the vaccination coverage by the dental students.

Nevertheless, this systematic review highlights the overall low vaccination estimates among Indian dental students. Despite widespread public health vaccination campaigns in India, vaccination coverage are low, which calls for urgent action. Lamichhane et al. (2025) in their systematic review of vaccination coverage among medical students suggested multiple strategies, such as awareness programs, ready availability, subsidization or free vaccination, mandatory vaccination, observing vaccination week, incentives, regular reminders, monitoring and follow-up can be implemented at institutional levels to improve the vaccination coverage. Mandatory institutional HBV policy and documentation of antibody titers would increase the acceptance of vaccination as they cannot participate in clinical training. This is required owing to the high risk of dental procedures and to safeguard both students and patients.

The findings from this analysis emphasize the need for mandatory HBV policy among dental students. Implementation of such policy requires consideration of multiple factors like feasibility and implementation mechanisms within the institutional framework as it would require structured documentation, follow-up, integration of verification systems into admission process and periodic audits. Potential challenges include institutional autonomy, workforce, and financial resources across various dental colleges may influence the sustainability of the implementation.

## 5 Conclusion

Complete HBV coverage among Indian dental students was suboptimal, with a pooled prevalence of 53.9%. Dental students who had received fewer than three doses and those who had never been vaccinated represent distinct groups and were not quantified in this review. Age and gender distribution were not significantly associated with complete HBV coverage, whereas clinical dental students demonstrated higher complete HBV coverage than the estimates of studies from both preclinical and clinical dental students. The observed negative association with publication year raises concerns regarding complete HBV coverage over time. These findings highlight the need for dental institutions and regulatory bodies to strengthen HBV initiatives, including educational programmes, vaccination status monitoring, and institutional vaccination policies. However, the effectiveness of these interventions were not evaluated in the included studies and warrants further investigation.

## Consent to participate declaration

Not applicable.

## Artificial intelligence (AI) use disclosure statement

The author(s) used ChatGPT: GPT-5.5; OpenAI, San Francisco, CA, USA for Language editing and grammatical correction and text refinement and summarization.

## CRediT authorship contribution statement

**Deepika Chenna:** Writing – review & editing, Formal analysis, Data curation, Conceptualization. **Abhinav N. Tadikonda:** Writing – review & editing, Formal analysis, Conceptualization. **Mithun Pai:** Writing – original draft, Data curation, Conceptualization. **Maanissa Phukan:** Writing – original draft, Data curation, Conceptualization. **Danica Alicia Menezes:** Writing – original draft, Data curation, Conceptualization. **Kalyana C. Pentapati:** Writing – review & editing, Writing – original draft, Software, Methodology, Formal analysis, Data curation, Conceptualization.

## Consent for publication

Not applicable.

## Ethics approval and consent to participate

Ethical approval was not required for this study because it is a systematic review and meta-analysis based exclusively on data from previously published studies. No human participants or animals were directly involved, and no identifiable individual-level data were collected.

## Ethics declaration

Ethical approval was not required for this systematic review and meta-analysis, as it involved secondary analysis of data from previously published studies.

## Funding

None.

## Declaration of competing interest

The authors declare that they have no known competing financial interests or personal relationships that could have appeared to influence the work reported in this paper.

## Data Availability

All supporting data for this review are included within the manuscript.

## Appendix A Supplementary data

Supplementary material 1 PRISMA 2020 Checklist

Supplementary material 2 References of the included studies

Supplementary material 3 Search strategy for various databases
