# Supplementary material for: Hepatitis B vaccination coverage among Indian dental students – Systematic review and meta-analysis
Source: Prev Med Rep. 2026 Nov 1;71:103691. doi: 10.1016/j.pmedr.2026.103691 (PMC13755964; doi:10.1016/j.pmedr.2026.103691)
Supplement: Supplementary file 2 [file mmc2.docx]

References of the included studies:

[1]	Tirounilacandin P, Krishnaraj S, Chakravarthy K. Hepatitis-B infection: Awareness among medical, dental interns in India. Ann Trop Med Public Heal 2009;2:33–6.

[2]	Singh A, Purohit BM, Bhambal A, Saxena S, Singh A, Gupta A. Knowledge, attitudes, and practice regarding infection control measures among dental students in Central India. J Dent Educ 2011;75:421–7.

[3]	Vasanthakumar AH, D’Cruz AM. Awareness regarding hepatitis B immunization among preclinical Indian dental students. J Oral Heal Oral Epidemiol 2013;2:97–101.

[4]	Rathinavelu PK, Sai Pavithra R. Assessment of knowledge on occupational exposure to hepatitis B infection and vaccine among undergraduate students in a private dental college, Chennai. J Pharm Sci Res 2015;7:592–4.

[5]	Kumar S, Basak D, Kumar A, Dasar P, Mishra P, Kumar A, et al. Occupational Hepatitis B Exposure: A Peek into Indian Dental Students’ Knowledge, Opinion, and Preventive Practices. Interdiscip Perspect Infect Dis 2015;2015:1–6. https://doi.org/10.1155/2015/190174.

[6]	Monika Gayathri M, Santhosh Kumar MP. Knowledge, awareness and attitude among dental students about hepatitis B infection. J Pharm Sci Res 2016;8:168–70.

[7]	Santhosh Kumar MP. Knowledge, attitude and practices regarding needlestick injuries among dental students. Asian J Pharm Clin Res 2016;9:312–5.

[8]	Nagpal B, Hegde U. Knowledge, attitude, and practices of hepatitis B infection among dental students. Int J Med Sci Public Heal 2016;5:1123. https://doi.org/10.5455/ijmsph.2016.03102015170.

[9]	Pratha AA, Geetha R V. Awareness on Hepatitis-B vaccination among dental students-A Questionnaire Survey. Res J Pharm Technol 2017;10:1360. https://doi.org/10.5958/0974-360X.2017.00240.2.

[10]	Khandelwal V, Khandelwal S, Gupta N, Nayak UA, Kulshreshtha N, Baliga S. Knowledge of hepatitis B virus infection and its control practices among dental students in an Indian city. Int J Adolesc Med Health 2018;30. https://doi.org/10.1515/ijamh-2016-0103.

[11]	Sundar Ray S, Pattnaik S, Kumar Sharma A. Hepatitis B- A killer study of awareness and practice among dental students regarding Hepatitis B infection and prevention. J Evol Med Dent Sci 2017;6:6460–3. https://doi.org/10.14260/jemds/2017/1404.

[12]	Malhotra V, Kaura S, Sharma H. Knowledge, attitude and practices about hepatitis B and Infection Control Measures among dental students in Patiala. J Dent Allied Sci 2017;6:65–9. https://doi.org/10.4103/2277-4696.219977.

[13]	Ravoori S, Rosy SN, Bommireddy VS, Talluri D. Prevalence, Knowledge, Attitude, Practices of Needle stick Injuries during Clinical Training among Undergraduate Dental Students. J Indian Dent Assoc 2018;12:12–5.

[14]	Rathor KR, Lenka S, Dharmashree S, Sethy S, Dalai RP. Awareness, Mindset & Method Associated with Infection Control Measures in Dental Students. Indian J Public Heal Res Dev 2019;10:338–42. https://doi.org/10.5958/0976-5506.2019.03487.9.

[15]	Sharma A, Kolte R, Kolte A. Knowledge, Attitude and Behaviour among dental students concerning infection control measures. J Indian Dent Assoc 2019;13:13–6. https://doi.org/10.33882/jida.13.25165.

[16]	Breena KR, Monica G, Arora A. Knowledge and Awareness among Dental Students Regarding Hepatitis B Infection - A Cross Sectional Study. Int J Res Rev 2019;6:24–7.

[17]	Benarji KA, Anitha A, Suresh B, Aparna V, Praveena A, Penumatsa LA. Knowledge and attitude of dental students toward hepatitis B virus and its vaccination – A cross-sectional study. J Oral Maxillofac Pathol 2021;25:553. https://doi.org/10.4103/jomfp.jomfp_387_21.

[18]	Srinivasan A, Krishnakumar K, Shetty R, Tandale AB, Gulati S. Knowledge, attitude, and practice regarding hepatitis B infection among dental interns of a private dental college in Pune, India: A questionnaire study. J Oral Heal Oral Epidemiol 2022;11:74–80. https://doi.org/10.22122/johoe.v11i2.1242.

[19]	Shashikala R, Chowdhary A. Assessment of knowledge, attitude, practice and immune status towards Hepatitis B among Dental Students of Navi Mumbai. Pravara Med Rev 2024;16:67–73. https://doi.org/10.36848/PMR/2024/10000.510999.

[20]	Rami DS, Chauhan C, Sutaria BS. Infection Control Awareness and Practices among Dental Students in Gujarat: A Questionnaire Based Cross-sectional Study. J Clin Diagnostic Res 2024;18:ZC30–5. https://doi.org/10.7860/JCDR/2024/69165.19851.

[21]	Mohan S, Priyank H, Kumar G, Viswanath B. Knowledge, Attitudes, and Practices of Undergraduate Dental Students About Sterilization, Disinfection, and Infection Control: A Questionnaire-Based Study. Cureus 2024;16:e59525. https://doi.org/10.7759/cureus.59525.

[22]	Kashyap S, Manohar B, Bali AK, Sharma N, Dhiman M, Singh A, et al. Hepatitis B: Assessment of Knowledge, Attitude, and Practice among the Graduating Dental Students of Odisha: A Web-Based Survey. J Pharm Bioallied Sci 2024;16:S4390–2. https://doi.org/10.4103/jpbs.jpbs_792_24.

[23]	Baskaran S, Karthikeyan I, Subramanian V, Jagadeson M, Karthikeyan R, Revanth MP. Perception of dental students toward Hepatitis B and its vaccine - A cross-sectional study. J Glob Oral Heal 2025;8:43–8. https://doi.org/10.25259/JGOH_16_2024.

[24]	Dubey VK, Mirza Z, Vanshika V, Bashir U, Rani S. A cross-sectional survey of awareness, attitude, and preventive measures of occupational hazards by dental students and professionals in Faridabad city. J Dent Spec 2025;13:16–22. https://doi.org/10.18231/j.jds.2025.004.
