# Supplementary material for: Hepatitis B vaccination coverage among Indian dental students – Systematic review and meta-analysis
Source: Prev Med Rep. 2026 Nov 1;71:103691. doi: 10.1016/j.pmedr.2026.103691 (PMC13755964; doi:10.1016/j.pmedr.2026.103691)
Supplement: Supplementary file 3 [file mmc3.docx]

Search strategies: Hepatitis B vaccination among dental students.

| Database | Search strategy |
| --- | --- |
| PubMed/MEDLINE | ("Students, Dental"[MeSH Terms] OR "dental student*"[tiab] OR "dental undergraduate*"[tiab] OR "dental intern*"[tiab] OR "dentistry student*"[tiab] OR "BDS student*"[tiab] OR "dental college*"[tiab] OR "dental school*"[tiab]) AND ("Hepatitis B"[MeSH Terms] OR "Hepatitis B Vaccines"[MeSH Terms] OR "hepatitis B"[tiab] OR "HBV"[tiab]) AND ("Vaccination"[MeSH Terms] OR vaccin*[tiab] OR immuniz*[tiab] OR immunis*[tiab]) |
| Scopus | TITLE-ABS-KEY("dental student*" OR "dental undergraduate*" OR "dental intern*" OR "dentistry student*" OR "BDS student*" OR "dental college*" OR "dental school*") AND TITLE-ABS-KEY("hepatitis B" OR HBV) AND TITLE-ABS-KEY(vaccin* OR immuniz* OR immunis*) |
| EMBASE | ('dental student'/exp OR 'dental student*':ti,ab,kw OR 'dental undergraduate*':ti,ab,kw OR 'dental intern*':ti,ab,kw OR 'dentistry student*':ti,ab,kw OR 'BDS student*':ti,ab,kw OR 'dental college*':ti,ab,kw) AND ('hepatitis b'/exp OR 'hepatitis b vaccine'/exp OR 'hepatitis B':ti,ab,kw OR 'HBV':ti,ab,kw) AND ('vaccination'/exp OR 'vaccin*':ti,ab,kw OR 'immuniz*':ti,ab,kw OR 'immunis*':ti,ab,kw) |
| Dentistry & Oral Sciences Source | TX ("dental student*" OR "dental undergraduate*" OR "dental intern*" OR "dentistry student*" OR "BDS student*" OR "dental college*" OR "dental school*") AND TX ("hepatitis B" OR HBV) AND TX (vaccin* OR immuniz* OR immunis*) |
| CINAHL (EBSCOhost) | (MH "Students, Dental" OR TX ("dental student*" OR "dental undergraduate*" OR "dental intern*" OR "dentistry student*" OR "BDS student*" OR "dental college*")) AND (MH "Hepatitis B+" OR MH "Hepatitis B Vaccines" OR TX ("hepatitis B" OR HBV)) AND (MH "Vaccination+" OR TX (vaccin* OR immuniz* OR immunis*)) |
| Web of Science Core Collection | TS=("dental student*" OR "dental undergraduate*" OR "dental intern*" OR "dentistry student*" OR "BDS student*" OR "dental college*" OR "dental school*") AND TS=("hepatitis B" OR HBV) AND TS=(vaccin* OR immuniz* OR immunis*) |
